# Asenapine versus other atycipal antipsychotics for schizoaffective disorder Case study

**DOI:** 10.1192/j.eurpsy.2024.1452

**Published:** 2024-08-27

**Authors:** E. Shaska

**Affiliations:** Acute Services, Psychiatric Hospital, Vlora, Albania

## Abstract

**Introduction:**

Antipsychotics are psyschotropic medications that are indicated for the treatment of physchosis and mood disorders. Due to minimal side effects and their efficacy (affecting many receptors) compared to standard antipsychotics, today atypical antipsychotics are being used more and more as first-line treatment.

The aim of this study is to show the efficacy of asenapine and its tolerability as opposed to other atypical antipsychotics.

**Objectives:**

1. What are the side effects identified?
2. Side effects and efficacy of asenapine vs other atypical antipsychotics?

**Methods:**

It is a comparative, regular, clinical study, an examination case of a 53-year old female diagnosed with Schizoaffective Disorder 27 years ago and treated outside of hospital with atypical antipsychotics, such as: risperidone, olanzapine, quetiapine, aripiprazole, amisulpride. The study covers the timespan of 2010-2022.

**Results:**

The results showed that asenapine sublingual 15 mg had fewer side effects than other atypical antipsychotics. They were mouth dryness, headache, fatigue.

The other atypical antipsychotics caused: metabolic disorders, like considerable weight gain, cholesterol and glycaemia increase, extrapyramidal side effects, hyperprolactinemia.

Asenapine sublingual 15 mg was not as effective in treating Schyzoaffective Disorder as risperdal 5mg, olanzapine 15 mg, aripiprazole 20 mg, amisulpride 600 mg.

The efficacy of asenapine sublingual 15mg was the same as quetiapine 400 mg.

**Conclusions:**

This study showed that asenapine has minimal side effects but its efficacy in treating Schyzoaffective Disorder as monotherapy is lower than other atypical antipsychotics.

**Key words:**

antipsychotics, schyzoaffective disorder, side effects, efficacy

**Disclosure of Interest:**

None Declared

